# Prevalence of Abnormalities at Tandem Endoscopy in Patients Referred for Colorectal Cancer Screening/Surveillance Colonoscopy

**DOI:** 10.3390/cancers16233998

**Published:** 2024-11-29

**Authors:** George Triadafilopoulos

**Affiliations:** Department of Gastroenterology, Hepatology and Nutrition, University of Texas MD Anderson Cancer Center, 1515 Holcombe Blvd, Houston, TX 77030, USA; gtriadafilopoulo@mdanderson.org

**Keywords:** Screening for Barrett’s esophagus, screening for gastric cancer, endoscopy, colonoscopy, intestinal metaplasia, dysplasia

## Abstract

**Introduction:** Performing a tandem endoscopy and colonoscopy in selected individuals has advantages, such as the early detection of benign and/or precancerous foregut diseases; it is efficient, and it may allow added therapies. It may also have disadvantages, such as generating anxiety from false-positive screening, possible harm from further testing, and unproven cost-effectiveness. **Aims:** We aimed to examine the prevalence of foregut endoscopic and histologic abnormalities in subjects referred for screening/surveillance colonoscopy who also underwent a tandem endoscopy. We wanted to (1) assess implications for cancer detection, intervention, and surveillance of precancerous foregut abnormalities, (2) identify benign foregut lesions, and (3) generate data on the utilities of this tandem approach. **Patients and Methods:** A retrospective cohort study of consecutive subjects referred for screening or surveillance colonoscopy who also underwent an endoscopy. Based on national screening guidelines, responses to prompting questions, personal or family history, or other risk factors, subjects were assigned to tandem endoscopy with biopsies (modified Seattle and Sydney protocols), under one anesthesia. **Results:** Of the 1004 patients referred for colonoscopy, 317 (32%) underwent tandem endoscopy. There were 214 women and 103 men. There were 237 Whites, 16 Asians, 40 Blacks, and 24 Hispanics. Median age was 59 (range 19–85). At endoscopy, we identified actionable benign (45%) peptic, inflammatory, and *H. pylori*-related abnormalities, and premalignant findings (i.e., intestinal metaplasia, 27%, dysplasia, 2%, and cancer 0.9%), comparable to the premalignant (40.3%) and malignant (0.6%) colonoscopy yield. **Conclusions:** When implemented based on national screening guidelines, tandem EGD and colonoscopy combines Barrett’s esophagus and gastric cancer screening in one examination, and it has a high yield in a diverse US population.

## Simple Summary

This retrospective analysis of selected patients who underwent guideline-based, point of care, endoscopy in tandem with guideline-based, screening/surveillance colonoscopy reveals potentially significant foregut pathology (not necessarily precancerous). Potentially precancerous foregut pathology is as prevalent as precancerous colon polyps found at colonoscopy. Benign foregut endoscopic and histologic findings may be amenable to dietary and pharmacologic interventions. Tandem EGD combines Barrett’s esophagus and gastric cancer screening in one examination and should be considered in all subjects referred for screening or surveillance colonoscopy.

## 1. Introduction

Colonoscopy, the most common screening test for colorectal cancer (CRC), is associated with reduced CRC incidence and mortality through early detection and treatment of cancer [1]. Adenomas are found in nearly 40% of screening colonoscopies in the US, and after their removal, patients undergo future surveillance. Several genetic polyposis syndromes are associated with an increased risk of CRC or cancers elsewhere in the GI tract, calling for the use of endoscopy for screening and surveillance of gastric and or duodenal abnormalities [2,3,4]. Performing elective upper (EGD) and lower (Colonoscopy) endoscopic procedures on the same day could be a patient-centered and less costly approach than a two-stage approach performed on different days, when clinically appropriate.

Recent guidelines [5] and epidemiologic reviews [6,7] suggest a single screening endoscopy for patients with chronic GERD symptoms, and three or more additional risk factors for Barrett’s Esophagus (BE) [8]. A consensus statement recommends *Helicobacter pylori* (HP) testing and endoscopic screening with biopsies should be offered at age 50 to those with a family history of gastric cancer (GC) in a first-degree relative, first-generation immigrants from high GC-incidence regions, and those belonging to racial/ethnic groups at increased risk [9]. A recent guideline suggests that patients 60 years or older with dyspepsia be investigated with EGD [10,11].

Performing a tandem EGD and colonoscopy in selected individuals has advantages, such as the following: it allows for the early detection of benign and/or precancerous foregut diseases; it is covered by insurance, as long as it is based on national guidelines; it decreases the endoscopists’ time demand, and improves patient transportation logistics; it is well-tolerated; it potentially reduces anesthesia risks; it improves efficiencies (scheduling and endoscopy unit utilization); and it provides the benefit of added therapies (diet, proton-pump inhibitor use, and endoscopic or surgical treatments). However, this approach may have disadvantages, such as the following: there is an unknown prevalence of these conditions in the general US population, despite its increasing diversity; its findings may generate anxiety produced by false-positive screening; it may lead to harm associated with further diagnostic testing; and it may lead to overdiagnosis of conditions that may be treated but would never have become clinically apparent. Finally, it has unproven cost-effectiveness.

In this study, we aimed to examine the prevalence of foregut endoscopic and histologic abnormalities in patients undergoing endoscopy, based on national guidelines, in tandem with point-of-care screening/surveillance colonoscopy for CRC. For this, we followed a rigorous endoscopic protocol, involving white-light and NBI chromoendoscopy in conjunction with meticulous histologic assessment using the Sydney classification, the Seattle biopsy protocol, and histologic assessment of the squamocolumnar junction (SCJ) and the duodenum in all patients. We wanted to (1) assess the implications for early cancer detection and intervention, and surveillance of potentially precancerous abnormalities (i.e., intestinal metaplasia and dysplasia), (2) identify benign foregut lesions that would benefit from intervention (i.e., peptic pathology, celiac disease), and, ultimately, (3) generate pilot data on the utilities of the tandem approach in such select patients.

## 2. Patients and Methods

### 2.1. Patients

This retrospective cohort study of prospectively collected information was approved by the MD Anderson Institutional Review Board. No individual study consents were obtained, and all data were kept confidential in an encrypted database. Our population consisted of consecutive subjects who underwent screening or surveillance colonoscopy in our ambulatory surgery center by the author, from 1 February 2023 to 31 May 2024. All patients were referred for CRC screening or surveillance (not diagnostic) colonoscopy—as an absolute inclusion criterion—to the outpatient MD Anderson Endoscopy facility in Woodlands, TX, USA. Patients did not volunteer foregut symptoms, such as dyspepsia or acid reflux, but they potentially reported such symptoms upon questioning. Based on their responses to prompting questions, and their personal or family history, or other risk factors, the screening nurse assigned them an indication score and added an EGD, to be performed in tandem, under one anesthesia. Before proceeding with the procedures, informed consent outlining the risks, benefits, alternatives, and potential complications was obtained and documented. Inclusion criteria were mainly based on national guidelines for BE (score > 4) and GC screening (score > 1) (Table 1), or other conditions justifying EGD in tandem with colonoscopy (such as Lynch syndrome, family history of esophageal or gastric cancer, or alarm symptoms, such as dysphagia or early satiety, if elicited. The breakdown of the justification for EGD in tandem with colonoscopy, the potential ICD-10 codes used for insurance authorization, and number of patients enrolled by indications are listed in Table 2. There were no other exclusion criteria. Specifically, subjects with prior gastrointestinal surgery for benign (i.e., bariatric) or malignant (i.e., partial colectomy for cancer) disease, or medication use (i.e., proton-pump inhibitors, aspirin or NSAID use) were not excluded.

**Figure 1:**
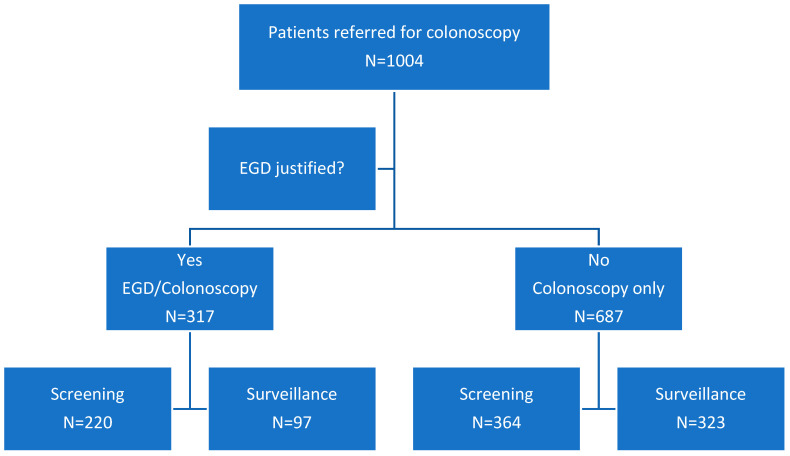
Study flow diagram.

During the study period, 1004 subjects underwent screening or surveillance colonoscopy by a single experienced endoscopist, using a standardized endoscopy and colonoscopy protocol, as described in detail in Methods (Appendix A). Of these, 317 subjects (32%) received tandem EGD and colonoscopy after having fulfilled the entry criteria justifying the EGD, and 688 only received colonoscopy. These two groups were then compared, based on the colonoscopy finding, to ensure that the dual procedure subjects were similar in terms of demographics, clinical indications for colonoscopy referrals, polyp burden, and related histology. Figure 1 depicts a flow diagram of the study populations and highlights clinical indications in each group.

### 2.2. Methods

After insurance clearance, patients received standard preparation for endoscopy and colonoscopy per MD Anderson guidelines, as needed, to reduce aspiration or bleeding risks. Specific procedural details are described in Appendix A.

## 3. Results

### 3.1. Patients and Overall Findings

**Figure 2:**
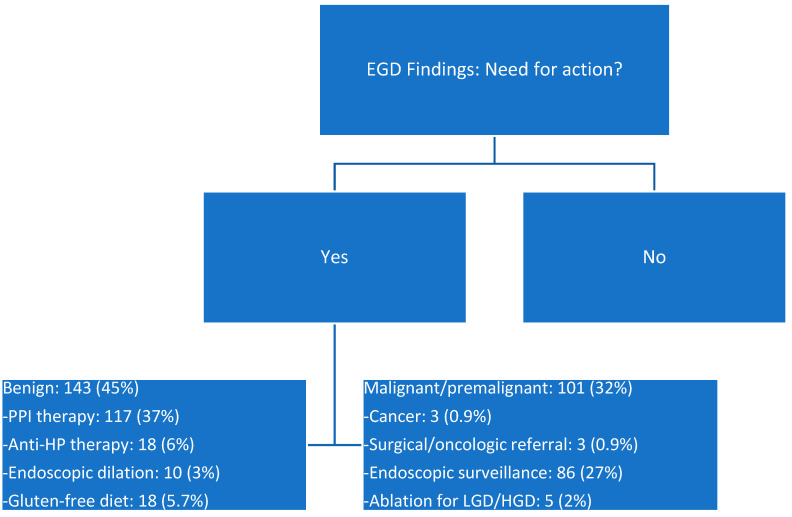
Need for action depending on endoscopic and histologic findings at endoscopy in the 317 subjects of the dual EGD/colonoscopy cohort.

Of the 317 subjects in the cohort who underwent tandem EGD and colonoscopy, there were 214 women (67.5%) and 103 men (32.5%). There were 237 Whites (74.7%), 16 Asians (5%), 40 Blacks (12.6%), and 24 Hispanics (7.5%). Their median age was 59 (age range 19–85). In contrast, of the 687 subjects undergoing only colonoscopy, there were 378 women (55%) and 308 men (45%). In this group, there were 520 Whites (75.6), 21 Asians (3%), 70 Blacks (10.1%), 68 Hispanics (9.9%) and 8 Other (1.2%). Their median age was 60 (age range 21–90). When comparing the colonoscopy findings between the two groups, the tandem EGD/colonoscopy one (*n* = 317) revealed 39 hyperplastic polyps (12.3%), 10 serrated polyps (3.1%), 118 tubular and tubulovillous adenomas (37.2%), and 2 cancers (1 colon and 1 rectal; 0.6%), while the colonoscopy-only group (*n* = 687) revealed 100 hyperplastic polyps (14%), 39 serrated polyps (5.7%), 338 tubular and tubulovillous adenomas (49.2%) and 10 colorectal cancers (1.4%). This analysis suggested that the colonoscopy was of superior quality in both groups, without any negative impact of the EGD on the colonoscopy polyp yield [12]. Figure 2 highlights the actionable findings on tandem EGD, and the actions taken at the discretion of the investigator.

Of the 317 subjects who underwent tandem EGD and colonoscopy, 6 (1.9%) had evidence of prior colocolonic or colorectal anastomosis, while 10 (3.5%) had prior ileocolonic anastomosis. Only two cases were incomplete (aborted) examinations, due to sigmoid strictures. Of the 687 subjects undergoing only colonoscopy, 46 (6.7%) had prior colocolonic, or colorectal anastomosis, while 26 (3.8%) had prior ileocolonic anastomosis. There were four subjects (4.6%) who had prior ileocolonic and colocolonic anastomosis, and one had colonoscopy through sigmoid colostomy. Only one case was an incomplete (aborted) examination, due to sigmoid stricture. At EGD, 20/317 patients (6.3%) revealed postoperative changes; as a result, select protocol biopsies could not be obtained. Ten had prior sleeve gastrectomy; eight had prior gastrectomy with Roux-en-Y anastomosis, one had prior gastrectomy with Billroth II anastomosis and prior esophagogastric anastomosis for proximal benign gastric tumor.

### 3.2. Endoscopy and Biopsies

**Figure 3:**
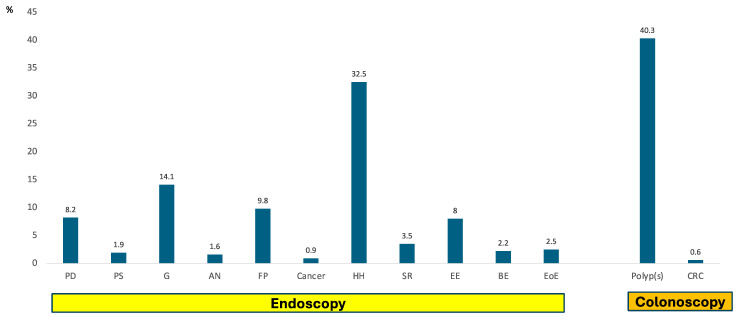
Point % prevalence of key benign and malignant endoscopic abnormalities at tandem EGD and colonoscopy in 317 subjects. PD: Peptic duodenitis; PS: Pyloric Stenosis; G: Gastritis; AN: Antral Nodule; FP: Fundic Polyp; Cancer; HH: Hiatal Hernia; SR: Schatzki’s Ring; EE: Erosive Esophagitis; BE: Barrett’s Esophagus; EoE: Eosinophilic Esophagitis; Polyps; CRC: Colorectal Cancer.

**Figure 4:**
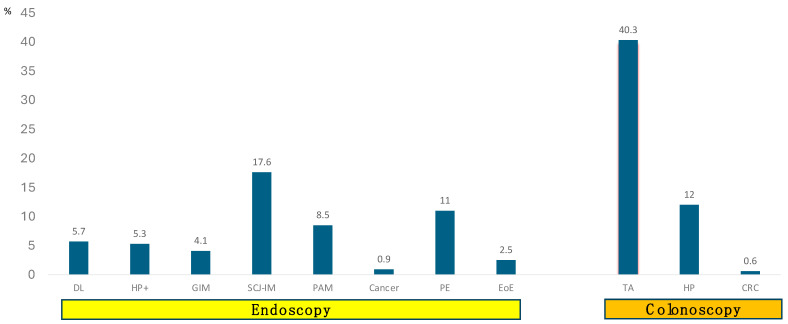
Point % prevalence of key histologic abnormalities at tandem EGD and colonoscopy in 317 subjects. DL: Duodenal lymphocytosis; HP+: Active *H. pylori* infection; GIM: Gastric Intestinal Metaplasia; SCJ-IM: Squamocolumnar Junction–Intestinal Metaplasia; PAM: Pancreatic Acinar Metaplasia; Cancer; PE: Peptic esophagitis; EoE: Eosinophilic esophagitis; TA: Tubular Adenoma (including serrated polyps); HP: Hyperplastic polyps; CRC: Colorectal cancer.

Duodenum: Per-protocol endoscopic assessment of the duodenum revealed peptic duodenitis in 23 patients; only 6 were associated with histologic erosive duodenitis. One patient had an *H. pylori*-negative bulbar ulcer. Two patients exhibited post-bulbar duodenal strictures (all dilated using the endoscope). In total, 26 patients (8.2%) were classified as peptic duodenitis. All these patients were treated with oral omeprazole, with 20 mg daily for 2 months. The remaining had normal duodenal appearance. Protocol bulbar and post-bulbar biopsies of the duodenum revealed 18 cases of duodenal lymphocytosis, 3 of which had associated villous blunting, consistent with celiac disease (CD). All were treated with gluten-free diet (Figure 3 and Figure 4).

Pylorus: Six patients (1.9%) were noted to have subtle benign pyloric stenosis, which was dilated using the endoscope, followed by treatment using oral omeprazole 20 mg daily for 2 months. No distinct pyloric ulcer was seen; no biopsies were obtained from the pylorus.

Antrum: Endoscopically, the antrum showed non-specific gastritis in 45/317 patients (14.1%). Of those, 14 were infected with *H. pylori* and they were all treated with triple therapy. Antral nodularity was seen in five patients (1.6%). There was no relationship between *H. pylori* infection and visible gastritis. Random (protocol) antral biopsies revealed gastric intestinal metaplasia GIM (one with dysplasia and one related to *H. pylori*) in 20 subjects (Figure 3 and Figure 4).

Incisura: Endoscopically, the incisura showed non-specific gastritis in 42/317 patients (13.2%). Of those, 14 were infected with *H. pylori* and they were all treated with triple therapy (the same as above). There was no relationship between *H. pylori* infection and visible gastritis. Random (protocol) incisura biopsies revealed GIM (one with dysplasia, and one related to *H. pylori*) in seven subjects. In addition, select incisura biopsies revealed fibrosis (*n* = 3), hemorrhage (*n* = 1) and autoimmune metaplastic atrophic gastritis (AMAG) (*n* = 3) (Figure 3).

Gastric body: Endoscopically, the gastric body showed non-specific gastritis in 20/317 patients (6.3%). Of those, 17 (5.3%) were infected with *H. pylori* and they were all treated with triple therapy. There was no relationship between *H. pylori* infection and visible gastritis. Random (protocol) gastric body biopsies revealed GIM (one with dysplasia, and one related to *H. pylori*) in 14 subjects (Figure 4). Select biopsies revealed fibrosis (*n* = 3), hemorrhage (*n* = 1), atrophy (*n* = 2), neuroendocrine cell hyperplasia (*n* = 1), and autoimmune metaplastic atrophic gastritis (AMAG) (*n* = 6). Fundic polyps (FPs) were seen and confirmed benign histologically in 31 cases (9.8%) (Figure 4).

Cardia: Endoscopically, the cardia revealed infiltrative tumor in three patients (two gastric, and one esophageal; both were confirmed histologically). It was normal in 314 cases. Protocol biopsies were obtained universally 0.5 cm below the SCJ. In these, 129 (41%) showed chronic inflammation (12 with histologic *H. pylori* infection); GIM was found in 14 (4.4%) of subjects (none related to *H. pylori* infection). Parietal cell hyperplasia was noted in nine cases, and atrophy and fibrosis in three. Pancreatic acinar metaplasia (PAM) was seen in six cases (1.9%). Sliding hiatal hernia was noted in 103/317 subjects (32.5%) and was associated with Schatzki’s ring in 4 patients. Overall, 11 subjects (3.5%) exhibited Schatzki’s ring; one required dilation (Figure 3 and Figure 4).

Squamocolumnar junction: Endoscopically, the SCJ showed two cancers (one gastric and one esophageal, confirmed histologically, see above) and peptic esophagitis extending upwards to the distal esophagus in 25/317 subjects (8%).

Twenty-four patients had erosive esophagitis in four quadrant biopsies of the SCJ (7.6%) and two had histologic ulcers. All were treated with oral omeprazole, with 20 mg daily for 2 months. Fifty-six subjects were found to have IM at the SCJ (17.6%), variably associated with GIM elsewhere (see below); four had LGD and one HGD, and were referred for endoscopic eradication therapy. There were 21 cases of PAM (6.6%), 3 of them occurring in conjunction with IM (but no dysplasia); if cardiac PAM was added, which was seen in six cases (1.9%), the total % prevalence of PAM in that region is 8.5% (Figure 3 and Figure 4).

Esophagus: Endoscopically, the tubular esophagus revealed peptic esophagitis in 25/317 subjects (8%). These were distributed as the following: 13 LA-Grade A; 15 LA-Grade B; 2 LA-Grade C and 5 LA-Grade D. There were seven patients with endoscopically visible Barrett’s esophagus (BE), five containing dysplasia. All were treated with oral omeprazole, with 20 mg daily for 2 months. Histologically, there were eight cases of eosinophilic esophagitis. There were nine patients with histologic erosive esophagitis on distal esophageal biopsies and 26 patients had histologic changes of GERD with papillary hyperplasia and chronic inflammation without erosions or ulcers, totaling 35 (11%) of patients (Figure 3 and Figure 4). All were treated using daily omeprazole, with 20 mg for 2 months. One patient had esophageal *Candidiasis* and was treated with fluconazole 100 mg orally for 10 days.

Combination findings: Supplementary Figure S1 depicts a diagram of biopsy protocol at endoscopy. In addition to station biopsies, focal, endoscopic abnormalities were separately biopsied or resected. Immunohistochemistry for *H. pylori* infection was obtained in all cases. Based on the focal (isolated), or contiguous presence of GIM of the antrum, incisura, gastric body, and cardia, as well as IM of the SCJ, 86/317 subjects (27%) were recommended endoscopic surveillance, 3 years after endoscopy (Supplementary Figure S2). In addition, 5/86 cases contained low- or high-grade dysplasia at SCJ and were referred for endoscopic eradication therapy (EET) (Figure 2).

## 4. Discussion

In this study of select patients referred for screening or surveillance colonoscopy who also underwent a tandem EGD (32% of total cohort), we identified actionable benign (45%), premalignant (31%), and malignant (0.9%) findings that are comparable to the precancerous polyp (40.3%) and malignancy (0.6%) yield of colonoscopy. Tandem endoscopy combines BE and GC screening in one examination and is practically and fiscally attractive, with the potential for saving and improving lives. Thus far, screening for BE is considered separately in Western, mostly Caucasian, populations with GERD, and screening for GC is considered separately in Asian and Hispanic populations with high incidence of *H. pylori* infection, without considering both concomitantly, thereby potentially maximizing the cost-effectiveness of future surveillance. Our findings validate the national guidelines [5,6,7,8] recommending screening for EGD, and they provide additional justification by identifying actionable benign endoscopic and histologic abnormalities. Further, substantiated by rigorous and hitherto never-implemented histologic assessment, our study provides valuable histologic information on the prevalence of precancerous abnormalities, such as intestinal metaplasia, with resulting implications for cancer screening and surveillance.

Same-day upper and lower endoscopy has many potential advantages, by reducing costs, shortening hospital stay, and expediting patient care, but its yield and cost-effectiveness remain unclear [13]. The benefits of performing an endoscopy must be balanced against its potential harms, and such practice should follow society guidelines [14]. Inappropriate use of upper endoscopy exposes patients to unnecessary risks and financial burden. A meta-analysis of 53,392 patients identified a high frequency of inappropriate indications for upper endoscopy and a higher diagnostic yield in patients who had an appropriate indication [15]. Physician offices and ambulatory surgery centers have much higher different-day procedure rates compared with hospital outpatient departments [16], representing an opportunity for quality improvement and financial savings. The sequence (upper–lower) or lower–upper of same-day double gastrointestinal endoscopy does not affect total procedure time or medication use [17].

GERD symptoms are an essential criterion for BE and EAC screening, but they are not universally adopted. A systematic review and meta-analysis examined the prevalence of BE/EAC in those with and without GERD [18]. The overall risks for BE and long-segment BE are higher in patients with GERD, but the risk for short-segment BE did not differ between the two groups. In nine population-based studies (2244 patients with and 3724 patients without GERD), BE prevalence in patients without GERD was 4.9% (95% CI, 2.6–9%). BE prevalence was highest in North American studies (10.6% [GERD] and 4.8% [non-GERD]). Given the substantial BE prevalence in those without GERD, future BE/EAC early-detection guidelines may need revision.

It is important to examine to what degree our endoscopic and histologic findings were clinically significant (actionable), innocuous, or harmful. Duodenal lymphocytosis (DL), noted in 5.7% of our cohort, is a non-specific histologic alteration of unknown cause, at times associated with dyspepsia, as it was in 17% of our cases. DL corresponds to Marsh grade 1, that is, mild inflammation of the duodenal mucosa, with no crypt hyperplasia or villous atrophy. About 11% of DL patients exhibit clinical and serologic features of gluten sensitivity. In our cohort, three subjects had villous blunting, suggestive of CD. *Helicobacter pylori* gastritis may also contribute to DL, which may resolve following eradication, but it was not seen in our patients [19]. In all our cases of DL, a gluten-free diet was implemented (Figure 3).

Active *Helicobacter pylori* infection, seen in 6% of our cohort (Figure 3), denotes a risk of chronic gastritis, peptic ulcer disease, and gastric neoplasia. Because most infections remain clinically silent, *H. pylori* case-finding thus far is based on symptoms (i.e., dyspepsia), or high-risk indicators, such as racial or ethnic background and family history, like in our cohort. However, this approach misses many infected individuals who remain at risk of GC, hence our use of the Sydney protocol. Individuals with chronic *H. pylori* are at risk of gastric preneoplasia, which is also asymptomatic and only reliably diagnosed using an endoscopy and biopsy. Thus, to make a significant impact on gastric cancer prevention, a systematic approach is needed. *H. pylori* eradication must also be optimized, given sharply decreasing rates of success with common therapies and increasing antimicrobial resistance [20]. All our identified *H. pylori* subjects were treated with triple therapy and were confirmed eradicated by stool antigen testing 2–3 months after therapy, without any evidence of antimicrobial resistance. Those with gastric pre-neoplasia—HP-related or not—were then entered into endoscopic surveillance.

Gastric intestinal metaplasia (GIM), be it of samples of endoscopic abnormalities (i.e., nodules) or randomly, using the Sydney protocol, was noted in 40 (26%) of our cohort (Figure 4 and Figure S2). Because of its risk for gastric neoplasia, particular attention was paid to identify the concomitant *H. pylori* infection and treat it, but also to exclude dysplasia. Regardless, all patients with GIM were entered into endoscopic surveillance in 3-year intervals (Figure 3)**.** AMAG, found in 10 patients (3.1%) of our cohort, is an inherited autoimmune disease and has a 3-fold risk of gastric adenocarcinoma. A surveillance endoscopy every 3 years should be considered in individuals with atrophic gastritis, based on anatomic extent and histologic grade, as it was done in all our cases [21].

Fundic polyps (FPs) are common (9.8% in our cohort) and benign, with little malignant potential. Their prevalence has been increasing with the widespread and frequent use of PPIs. Most FPs occur in the gastric body, are small (<5 mm) and sessile, and surveillance is not required. However, histopathology is necessary if endoscopic findings different from ordinary FPs are noted [22]. In all our cases, representative FPs were biopsied to confirm histology; there were no polyps in our cohort with disturbing endoscopic characteristics or size that required polypectomy.

Careful visualization of the cardia in antegrade and retrograde views was universally performed and combined with endoscopic assessment for hiatal herniation. Universally, biopsies were obtained 0.5 cm below the squamocolumnar junction (SCJ) to assess for GIM and its possible link to GIM of the stomach, gastric atrophy, and SCJ-IM. Intestinal metaplasia in the esophagus (Barrett’s esophagus IM, or BE-IM) and stomach (GIM) are considered precursors for esophageal and gastric adenocarcinoma, respectively. Studies have revealed the gradual molecular and phenotypic transition from a gastric to intestinal phenotype (IM) in the esophagus and stomach. Because BE-IM and GIM can predispose to cancer, this new understanding of a common developmental trajectory leads to a more unified approach to detection and treatment [23]. Indeed, this concept served as an initial thrust to this clinical study, namely, the assessment of prevalence and further phenotypic characterization of these regions during one endoscopic procedure. Although short-segment gastric metaplasia (SS-GM) is a distinct entity from short-segment intestinal metaplasia (SS-IM) and the malignant potential of GM is lower than IM, a thorough pathology and endoscopy review has questioned whether SS-GM warrants inclusion in BE surveillance [24]. We did not encounter any cases of SS-GM in our cohort.

SCJ-IM is a common finding in GERD, and it may represent an early step in the development of esophagogastric junction adenocarcinoma (EGJAC) in the West. In our cohort, 17.6% exhibited SCJ-IM, some 2.2% associated with short-segment BE, others with GIM of the cardia (1.6%) or the stomach. Worldwide, SCJ-IM may represent progression along the Correa cascade triggered by *Helicobacter pylori*. Using decision analytic modeling that evaluated the cost-effectiveness of endoscopic surveillance of EGJ-IM, low-intensity longitudinal surveillance (every 5 years) was cost-effective in populations with higher EGJAC incidence [25]. Aiming at estimating lifetime benefits, complications, and cost-effectiveness of GIM surveillance using EGD, another study concluded that surveillance of incidentally detected GIM every 5 years is cost-effective [26]. These models unfortunately address distinct but overlapping issues, those of EAC, EGJAC and GC, without considering EGD as the sole tool for clinical exploration. It is our hope that, particularly in the modern era of mass migration and globalization, this pilot study will initiate further real-world studies evaluating the impact of screening/surveillance on the combined incidence and mortality of foregut cancers and their ominous implications. Indeed, in our limited cohort, three EGJACs were noted, only one reporting dysphagia and two reporting dyspepsia, upon questioning prior to colonoscopy.

PAM and IM overlap infrequently at the gastroesophageal junction/distal esophagus (GEJ/DE). A cohort study suggested that PAM at the GEJ/DE is associated with protective effect against IM [27]. In our cohort, 8.2% were noted to have PAM of the cardia (*n* = 6) and at the SCJ (*n* = 20) alone, or in conjunction with IM (*n* = 3) but inadequate to make any claims of clinical importance. Nevertheless, in the context of larger prospective assessments, particularly using immunohistochemical studies for p53, the role of PAM will be further clarified.

Given the low prevalence of *H. pylori* in our cohort, we suspect that aspirin and NSAIDs, used by approximately 11% of the U.S. population [28,29], contributed to peptic lesions and the occurrence of GERD symptoms and dyspepsia. Overall, tandem EGD identified and allowed PPI treatment in 37% of patients, comprising benign peptic (gastro-duodenal and esophageal) lesions, both for symptoms and prevention of complications. In addition, anti-HP therapy was given in 18 patients (6%), while endoscopic dilation was applied in 10 patients (3%) (Figure 3).

Figure 2, Figure 3 and Figure 4 highlight the benefits of the tandem approach. Collectively, the identification of actionable benign (45%) and malignant or premalignant (32%) lesions at EGD, matched those with actionable benign (12%) and premalignant polyps (40.3%) and CRC (0.6%) at colonoscopy. We believe that the yield of important pathology in our cohort—carefully selected on meeting screening criteria for BE, GC, or both—together with history of inherited polyposis syndromes, family history of esophageal and gastric cancer, and dysphagia and early satiety, amply justify the tandem approach. We did not identify any actionable foregut pathology in our 18 patients with inherited polyposis syndromes.

Our dual same-day procedure approach has a multidimensional impact on institutional resources, due to the added endoscopy personnel, and facility utilization, increasing by 15–30 min per patient. The fiscal impact, through identification of foregut pathology, will generate billing from surgical pathology, additional billing for physician services and downstream billing from imaging, including EUS, or downstream billing from surgical and oncology services. All these will need to be considered and justified, prior to a general implementation of this approach to ensure cost-effectiveness, but it is beyond the scope of our pilot effort. There is also a research impact: based on preliminary data, we could examine the role of additional screening modalities, currently approved (i.e., TissueCypher), participate in trials of other new screening modalities (i.e., liquid biopsies), and possibly capitalize on meticulously collected biopsies acquired for basic research endeavors on disease genetics, proteomics, etc., using microdissection [30].

There are limitations in this study that could be addressed prospectively, based on formally collected clinical research forms (CRFs) by uniformly trained and operating clinical coordinators, more accurately soliciting information, not only based on the BE and GC screening criteria (or both), but also on standardized semiquantitative symptom questionnaires, more elaborate family history information, and on a larger and more racially and ethnically diverse patient population presenting for colonoscopy to multiple endoscopists, with variable expertise in recognition and endoscopic sampling and removal of both foregut and colonic pathology. The data procured in this study were limited to an outpatient sample who were ASA class I-II, with insurance coverage, seen at a suburban outpatient endoscopy facility, and potentially not representing the larger metropolitan Houston area in diversity, attitude, and discipline towards cancer screening and surveillance. Further, a uniform histopathologic interpretation of precancerous and malignant lesions, preferably by two independent pathologists (plus a referee), enhanced by immunohistochemical and molecular analyses, would provide more precise insights. By integrating cutting-edge imaging technology with advanced software, digital pathology may improve quality assurance and standardization. Artificial intelligence significantly improves cancer diagnosis, classification, and prognosis. It also enhances the spatial analysis of the tumor microenvironment and enables the discovery of new biomarkers, advancing their translation for therapeutic applications [31]. Finally, actions taken in this cohort were solely based on one physician’s attitude, not representing a collective, protocol-driven clinical management of actionable findings; this could only be done in a prospective, multi-site, collaborative effort. Nevertheless, this is a real-life study providing intriguingly promising information on foregut/hindgut screening and surveillance, not heretofore properly addressed.

## 5. Conclusions

In conclusion, our analysis of selected patients who undergo guideline-based, point-of-care endoscopy, in tandem with screening or surveillance colonoscopy reveals significant foregut pathology (not necessarily precancerous), and merits formal prospective study. Our approach identifies potentially actionable foregut pathology that is almost as prevalent as precancerous colon polyps and cancer. Beyond further endoscopic surveillance for preneoplasia, benign foregut endoscopic and histologic findings may be amenable to other interventions. Yet, the long-term implications and cost-effectiveness are uncertain, and will require further study.

## Figures and Tables

**Table 1:** Guideline-based criteria for BE and GC screening.

Screening Criteria for BE	Yes (1); No (0)	Screening Criteria for GC	Yes (1); No (0)
Chronic heartburn and regurgitation		First-degree relative with GC	
Male		First-generation immigrants from high *H. pylori*-prevalence regions	
Age > 50		African Americans, Alaskan Natives, American Indians, Asian Americans, and Hispanic Americans	
White		Age > 60 with dyspepsia *	
Tobacco			
Obesity (BMI > 30)			
**Total**	**EGD if score > 4**	**Total**	**EGD if score > 1**

* Dyspepsia: A persistent or recurrent pain, or discomfort in the upper abdomen.

**Table 2:** Justification for EGD in the study cohort.

Indication	Potential ICD-10 Codes Used	Number of Patients
Criteria for BE screening met (Score > 4)	Z13.810; K21.9; R13.10	132
Criteria for GC screening met (Score > 1)	Z13.810; K30	86
Combined BE and GC criteria	Z13.810; K30; K21.9	38
Inherited polyposis syndrome (Lynch, JPS, FAP)	D13.91; Z15.09; D12.6	18
FH esophageal or gastric cancer	Z80.0	25
Miscellaneous alarm symptoms (dysphagia, early satiety)	R13.10; R68.81	18

## Data Availability

The original contributions presented in the study are included in the article/supplementary material, further inquiries can be directed to the corresponding authors.

## Supplementary Materials

The following supporting information can be downloaded at: https://www.mdpi.com/article/10.3390/cancers16233998/s1, Figure S1: Diagram of biopsy protocol at endoscopy. In addition to station biopsies below, focal, endoscopic abnormalities were separately biopsied or resected. Immunohistochemistry for *H. pylori* infection was obtained in all cases. Figure S2: Histologic % prevalence of intestinal metaplasia (IM) at selected locations of the stomach and squamocolumnar junction, potentially predisposing to GC and EAC. A total of 86 subjects (27%) were enrolled in endoscopic surveillance because of the cumulative prevalence of focal and/or scattered IM in the stomach and SCJ.

## Appendix A

### Appendix A.1. General

Pre-anesthesia assessment and clearance, standard history and physical examination, and measurement of BMI were also performed. Patients then underwent white-light and NBI cap-assisted endoscopy and white-light, NBI, AI- and cap-assisted colonoscopy. If at the time of endoscopy an endoscopic dilation was deemed necessary, it was performed using through-the-scope (TTS) balloons. Upon completion of the examinations and review of histology, patients were counseled accordingly, with implementation of pharmacologic therapy (if needed) for benign disease, further endoscopic eradication therapy, surgical or oncologic referral, or periodic surveillance (by endoscopy, colonoscopy, or both). Data entry was prospectively performed weekly, using Excel for subsequent retrospective review and analysis.

### Appendix A.2. Upper Endoscopy (EGD) and Biopsies

The goal of the EGD was to adequately identify and characterize mucosal abnormalities along the entire length of the esophagus, stomach, and duodenum. Adequate mucosal visualization was universally achieved after aspiration of luminal contents, full insufflation to expand the mucosal folds, and mucosal cleansing using irrigation with dilute simethicone via the working channel, as necessary. The Olympus high-definition white-light endoscopy (HD-WLE) system with image enhancement by narrow-band imaging (NBI; Olympus Medical Systems, Tokyo, Japan) was used. A minimum total EGD duration of longer than 9 min was allocated in all cases. All subjects received intravenous propofol under the supervision of an anesthesiologist or nurse anesthetist, unless there was concern regarding the risk of aspiration; in such cases, orotracheal intubation was performed. As a minimum, photo-documentation with at least one representative photograph of the esophagus/cardia with visualization of the squamocolumnar junction (SCJ) and the gastroesophageal junction, gastroesophageal junction/fundus in retroflexed view, body and antrum in anterograde view, incisura in retroflexed view, and distal extent of examination in the duodenum, in addition to any photo-documentation of suspicious abnormalities was performed. Standard terminology and classification systems, such as the Los Angeles classification for erosive esophagitis, the Prague classification for Barrett’s esophagus, the Paris classification for superficial neoplastic lesions, and the Eosinophilic Esophagitis Endoscopic Reference System were universally used [32]. Hiatal hernias were classified as sliding (small, medium, large), para-esophageal, or mixed. Special air insufflation views were also obtained in the distal esophagus to examine for the presence of Schatzki’s rings (SRs).

During EGD, the following biopsy protocol (Figure S1) was prospectively followed: at least six biopsy (in total) specimens from the distal, mid, and proximal esophagus were taken to examine for eosinophilic and reflux esophagitis, in addition to four-quadrant biopsy specimens for every 1–2 cm of visible (salmon-colored mucosa) BE (Seattle protocol), along with targeted biopsy specimens of mucosal abnormalities, such as suspicious areas of squamous dysplasia and inlet patch (minimum three separate bottles for distal, mid and proximal esophagus, plus targeted biopsies). These multilevel biopsies aimed to mainly improve the yield of histologic changes in eosinophilic esophagitis. If visible BE was noted, at least eight biopsy specimens were taken, even if patients had only 1–2 cm of BE, to increase intestinal metaplasia and dysplasia yield. In addition, and against conventional recommendations, routine four-quadrant biopsy specimens were also obtained straddling the normal or irregular SCJ (one bottle). Routine two-quadrant biopsy specimens were also separately obtained 0.5 cm below the SCJ (cardia) (one bottle).

To thoroughly assess the stomach, and irrespective of symptoms such as dyspepsia or reflux, or the use of acid-suppressing drugs, at least eight biopsy specimens from the following locations were obtained (based on the updated Sydney System biopsy protocol): four from the antrum (within 2–3 cm from the pylorus, two from the incisura angularis, and two from the body (one from the lesser curvature, about 4 cm proximal from the angle, and one from the greater curvature, about 8 cm distal to cardia). These separate antrum and gastric-body biopsy specimens allowed for assessment of extent, severity, and etiology of gastric atrophy and intestinal metaplasia. These biopsies were, as a minimum, separated into three jars (antrum, incisura, and body). Additional targeted biopsy specimens of focal abnormalities, such as polyps, were placed in separate jars. Specifically, all gastric ulcers were biopsied, irrespective of their appearance (i.e., shallow, or flat). If gastric ulcer biopsies were performed, then biopsy specimens were taken from the base and edges of the ulcer. *Helicobacter pylori* immunohistochemistry and histologic subtyping of gastric intestinal metaplasia (GIM) were also independently performed by the examining pathologists to improve the diagnostic and prognostic value of such gastric biopsy specimens. Gastric polyps were biopsied or preferably resected to establish a histologic diagnosis, particularly if they were solitary, for better accuracy, because histologic features may be patchy within a lesion. If there were multiple polyps, then the largest polyps were resected, and representative biopsies were taken from the remaining polyps. Samples of polyps were placed in separate jars. At least two bulbar and post-bulbar biopsies were universally obtained and placed in a separate container, to rule out celiac disease, since about 30% of cases may have normal endoscopic appearance. Additional lesions in the duodenum, such as nodules, were also biopsied and placed in separate containers. For purposes of endoscopic analysis, in addition to the prevalent cancers, erosive (peptic) duodenitis, duodenal ulcer, duodenal and pyloric stenosis, erosive gastritis, antral nodules, fundic polyps, hiatal hernia, Schatzki’s ring, erosive esophagitis (LA Stages A–D), eosinophilic esophagitis (EoE), endoscopic BE, and inlet patch were separately described and confirmed histologically [33].

The pathologic interpretation of biopsies was performed routinely by the assigned pathologist, who had access to the endoscopy report and endoscopic images corresponding to the tissue samples obtained. Clinical–pathological correlation was also made during weekly conferences that reviewed the endoscopic and histologic images only in a select number of cases, to address possible discrepancies on the finding or opinions, prior to further guidance to patients. In special occasions, additional pathologic assessments were made, involving the immunohistochemistry of select biopsy samples (i.e., p53, *H. pylori*). The pathological diagnosis of EoE was made when eosinophils were greater than, or equal to, 15 per high-power field (HPF), together with basal cell hyperplasia, elongation of papillae, superficial layering of eosinophils, extracellular eosinophilic granules, and fibrosis of the sub-epithelium [34]. Inflammatory cells in the squamous epithelium, basal zone hyperplasia and elongation of lamina propria papillae with capillary congestion were essential, but non-specific, features of reflux esophagitis [35]. The diagnosis of BE, as opposed to intestinal metaplasia of the squamocolumnar junction (SCJ-IM), was made upon demonstration of IM correlating with the endoscopic evidence of salmon-colored epithelium and extending to at least 1 cm of the length of the columnar mucosa, and containing either no dysplasia (ND), low-grade (LG)/high-grade (HG) dysplasia, or cancer [5]. For the histologic diagnosis of cancer, we endorsed the scheme of the International Gastric Cancer Association, which separates gastric cancers into type I, type II and type III, to represent the tumors at the distal esophagus, at the cardia and at the stomach distal to the cardia, respectively, further enhanced by molecular characterization, as needed [36]. The 2010 WHO classification, which recognizes four major histologic patterns of gastric cancers: tubular, papillary, mucinous, and poorly cohesive (including signet ring cell carcinoma), was used [37].

Potentially precancerous lesions with regard to gastric cancer, such as gastric polyps [38], gastric intestinal metaplasia (GIM), autoimmune metaplastic atrophic gastritis (AMAG), and gastric atrophy, were also characterized [39]. Active *H. pylori* infection was defined by the presence of histologic evidence of chronic gastritis (per location) and immunohistochemistry [40]. Peptic duodenitis [41] was histologically differentiated from the histological diagnosis of CD, consisting of an integrated assessment of (a) increased (25 T-lymphocytes/100 enterocytes) intraepithelial T-lymphocytes, also called duodenal lymphocytosis; (b) crypt hyperplasia; and (c) villous atrophy: a decrease in villous height, and alteration of the normal crypt/villous ratio (3:1) until the total disappearance of the villi [42]. Pancreatic acinar metaplasia (PAM) was defined as variably sized nests or lobules of acinar tissue located among, or at the base of, the mucosal glands. The tissue was composed of pancreatic acinar-like cells with abundant cytoplasm that was eosinophilic and granular in the apical and middle portions and basophilic in the basal area [43].

### Appendix A.3. Colonoscopy and Polypectomy

Screening and surveillance colonoscopy at MD Anderson Cancer Center and its satellites generally follow the AGA Clinical Practice Update on Risk Stratification for Colorectal Cancer Screening and Post-Polypectomy Surveillance [44]. Specifically, all individuals with a first-degree relative who was diagnosed with CRC, particularly before age 50, were considered at “increased” risk for CRC, while all individuals without a personal history of CRC, inflammatory bowel disease, hereditary CRC syndromes, other CRC predisposing conditions, or a family history of CRC were considered at “average” risk for CRC. Individuals at average risk for CRC are asked to initiate screening at the age of 45 years, while those at increased risk for CRC because of a first-degree relative with CRC initiate screening 10 years before the age at diagnosis of the youngest affected relative or at the age of 40 years, whichever is earlier. Risk stratification for initiation of CRC screening is generally based on an individual’s age, a known or suspected predisposing hereditary CRC syndrome, and/or a family history of CRC. The decision to continue CRC screening and post-polypectomy surveillance in individuals older than 75 years is typically individualized, based on an assessment of risks, benefits, screening history, and comorbidities. Other options, such as the fecal immunochemical test (FIT), the multitarget stool DNA fecal immunochemical test, and CT colonography for individuals at average risk for CRC are based on individual preference, and, if positive, they led to colonoscopy in our cohort.

Surveillance colonoscopy is based on high-risk criteria for future CRC following polypectomy revealing two or more premalignant polyps, including at least one advanced colorectal polyp, or five or more premalignant polyps. This cohort typically undergoes a one-off surveillance colonoscopy after 3 years. Post-CRC resection patients undergo a 1-year clearance colonoscopy, then a surveillance colonoscopy after 3 more years [45]. For patients with personal history of CRC who have undergone prior resection and anastomosis, the decision to pursue surveillance colonoscopy is left to the colorectal surgery team managing the patient, since many patients have variable previous interventions (including liver resection, chemotherapy, and/or radiation therapy). Such a decision is also based on the benefit to risk assessment due to age or comorbidity and evidence of disease recurrence, and whether it is loco-regional or metastatic.

Regardless, the ASA classification was strictly applied in our study population, allowing ASA class I and II subjects to undergo EGD and/or colonoscopy at our facility, and excluding those who were ASA class III (such as those with substantive functional limitations, one or more moderate-to-severe diseases, or morbid obesity (BMI ≥ 40), those with an implanted pacemaker, reduced ejection fraction, end-stage renal disease undergoing dialysis, and a recent history of cardiac or neurological events (unless cleared by cardiology). These subjects, as well as those with ASA class IV, were automatically referred to the main hospital endoscopy unit.

The goal of colonoscopy was to adequately identify and characterize mucosal abnormalities along the entire length of the colon, obtain biopsies, and remove all polyps. Adequate mucosal visualization was universally attempted, but not always achieved, after aspiration of luminal contents, full insufflation to expand the mucosal folds, and mucosal cleansing using irrigation via the working channel, as necessary. We performed colonoscopies during standard 30 min time slots. The standard bowel preparation was a split 4 L oral lavage of polyethylene glycol (PEG) electrolyte solution, except for rare patients with known previous intolerance. Four hours before the procedure they would have nothing by mouth, including water [46]. For the endoscopic assessment of colonic polyps and CRC, we used the Appearance, Classification, Enhanced Endoscopy and Safe Resection (ACES) algorithm that provides a systematic stepwise approach to the recognition and management of colon polyps, starting from visual impression suggestive of malignancy based on appearance, and looking for submucosal invasion using enhanced endoscopy to confirm the diagnosis, prior to a safe resection [47].

The endoscopy nurse recorded times for the following procedural events: colonoscope insertion into the rectum, identification of the base of the cecum, and withdrawal of the colonoscope across the anus, with times rounded to the nearest minute. The number, locations, and method of removal of polyps were likewise recorded. We defined colonoscopic insertion time as the time from insertion into the rectum to identification of the base of the cecum or ileocecal anastomosis and withdrawal time as the time from cecal or ileal identification to the time when the colonoscope was withdrawn across the anus (a minimum of 6 min). This withdrawal time did not include time taken for maneuvers, such as polypectomy, which were performed during the withdrawal phase of the examination. We used endoscopic estimates for the sizes of lesions at the time of colonoscopy [48]. As a minimum, photo-documentation with at least one representative photograph of the cecum, ileocecal valve, terminal ileum (if cannulated), each colonic segment and anorectum, surgical anastomoses, and all identified polyps, in addition to any other photo-documentation of suspicious abnormalities, was performed. If any mucosal abnormalities were seen beyond polyps, they were recorded and biopsied for histologic validation.

The histopathologic interpretation of biopsies was performed routinely by the assigned pathologist, who had access to the colonoscopy report and endoscopic images corresponding to the tissue samples obtained. Clinical–pathological correlation was also made during weekly conferences that reviewed the endoscopic and histologic images only in a select number of cases, to address possible discrepancies on the finding or opinions, prior to further guidance to patients. In special occasions, additional pathologic assessments were made involving the immunohistochemistry of select biopsy samples. Specifically, colon polyps were classified as inflammatory and hyperplastic (both non-precancerous), and the precancerous types of sessile serrated polyps (SSPs), tubular/tubulovillous adenomas (TA) with or without dysplasia), and colorectal cancer (CRC) [49].
