# Supplementary material for: Prevalence of Abnormalities at Tandem Endoscopy in Patients Referred for Colorectal Cancer Screening/Surveillance Colonoscopy
Source: Cancers (Basel). 2024 Nov 29;16(23):3998. doi: 10.3390/cancers16233998 (PMC11639759; doi:10.3390/cancers16233998)
Supplement: Supplementary file 1 [file cancers-16-03998-s001.zip › cancers-3296281-supplementary.pdf]

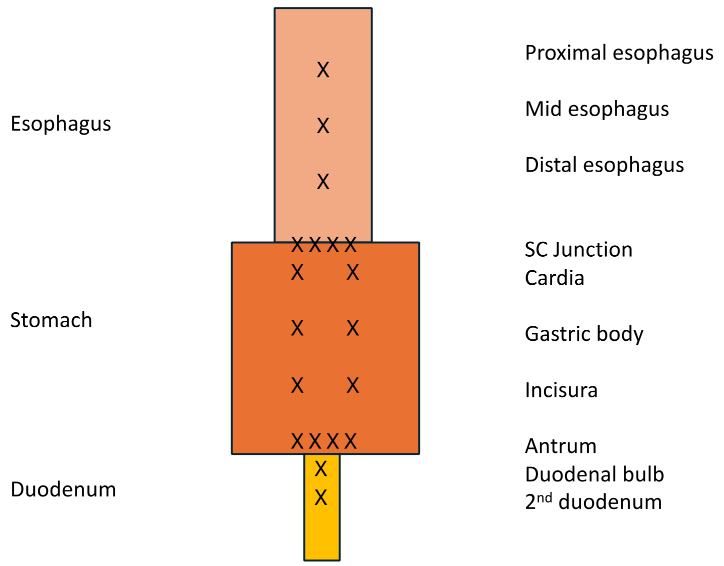

Supplemental Figure S1: Diagram of biopsy protocol at endoscopy. In addition to station biopsies below, focal, endoscooic abnormalities were separately biopsied or resected. Immunohistochemistry for *H. pylori* infection was obtained in all cases.

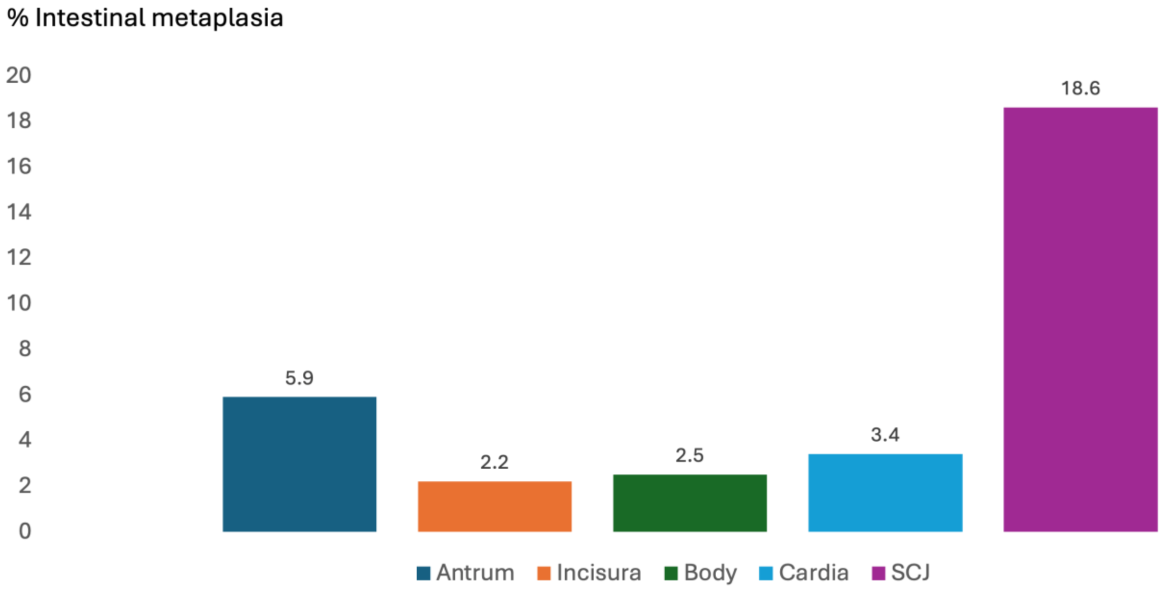

Supplemental Figure S2: Histologic % prevalence of intestinal metaplasia (IM) at selected locations of the stomach and squamo-columnar junction, potentially predisposing to GC and EAC. A total of 86 subjects (27%) were enrolled in endoscopic surveillance because of the cumulative prevalence of focal and/or scattered IM in the stomach and SCJ.
